# Retinal Vascular Density and Thickness in Long-Term Type 1 Diabetes Without Visible Vascular Signs of Retinopathy

**DOI:** 10.3390/jcm14041082

**Published:** 2025-02-08

**Authors:** Maria Sopeña-Pinilla, Marta Arias-Alvarez, Maria Isabel Lopez-Galvez, Elvira Orduna-Hospital, Guisela Fernandez-Espinosa, Ana Boned-Murillo, María Dolores Díaz-Barreda, Cristina Tomas-Grasa, Isabel Pinilla

**Affiliations:** 1 Department of Ophthalmology, Miguel Servet University Hospital, 50009 Zaragoza, Spain; mariasopenapinilla@gmail.com; 2 Aragón Health Research Institute (IIS Aragón), 50009 Zaragoza, Spain; martariasalvarez7@gmail.com (M.A.-A.); eordunahospital@unizar.es (E.O.-H.); guisela.fernandez3@gmail.com (G.F.-E.); anabomu@hotmail.com (A.B.-M.); lodiba92@gmail.com (M.D.D.-B.); 3 Department of Neurophysiology, Lozano Blesa University Hospital, 50009 Zaragoza, Spain; 4 Instituto Oftalmológico Fernández Vega, 33012 Oviedo, Spain; maribel@ioba.med.es; 5 Department of Applied Physics, University of Zaragoza, 50009 Zaragoza, Spain; 6 Department of Ophthalmology, Hospital Obispo Polanco, 44002 Teruel, Spain; 7 Department of Family Medicine, Lozano Blesa University Hospital, 50009 Zaragoza, Spain; cristina.tmgr@gmail.com; 8 Department of Ophthalmology, Lozano Blesa University Hospital, San Juan Bosco 15, 50009 Zaragoza, Spain

**Keywords:** diabetes mellitus, diabetic retinopathy, foveal avascular zone, retinal vascularization, optical coherence tomography angiography

## Abstract

**Background**: This research seeks to evaluate alterations in blood flow and structural features within the superficial (SCP) and deep (DCP) retinal capillary plexuses, as well as the choriocapillaris (CC), in patients with type 1 diabetes (DM1) who have no diabetic retinopathy (DR) over a period of 4 years. Additionally, the study examines changes in total and inner retinal thickness. **Methods**: A prospective, longitudinal analysis was conducted using optical coherence tomography (OCT) and OCT angiography (OCTA), involving 25 eyes from 25 DM1 patients with a disease duration of over 15 years. **Results**: A significant rise in vascular density (VD) was observed in the SCP, with no changes in foveal avascular zone (FAZ) metrics. Minimal changes were noted in the DCP and CC. Morphological abnormalities were frequent, but few changes were noted over time. No major differences were found in overall retinal thickness or inner retinal layers. There was a negative correlation between disease duration and VD in the temporal area of the SCP, as well as between disease duration and GCL++ parafoveal thickness (from the inner limiting membrane (ILM) to the outer limit of the inner plexiform layer (IPL)), along with a reduction in GCL++ perifoveal thickness. **Conclusions**: In DM1 patients without DR, the SCP VD tends to increase over 4 years, with no notable changes in retinal thickness.

## 1. Introduction

Diabetic retinopathy (DR) is a specific neurovascular complication of diabetic mellitus (DM) [1], a disease with a growing global prevalence [2]. It is recognized as the primary cause of blindness among the working-age population in developed countries [3].

Optical coherence tomography (OCT) angiography (OCTA) is a non-invasive tool used to visualize retinal capillary plexuses and choriocapillaris (CC) that has become crucial for the qualitative and quantitative evaluation of vessel changes in DR. OCTA is able to identify changes in the retinal microvasculature before the first signs of DR appear [4]. As the disease severity increases, more abnormalities can be found, including microaneurysms, ischaemia, and capillary dropout; intraretinal microvascular abnormalities; and neovascularization. Regarding possible quantitative modifications, OCTA can detect a reduction in vascular density (VD), modifications in the fractal dimension (FD), and changes in the foveal avascular zone (FAZ) parameters, such as its acircularity, area, and diameters.

DR begins with the appearance of microaneurysms, small haemorrhages, and/or lipoprotein exudates. Several studies have highlighted that patients with DM without visible clinical signs of DR often exhibit measurable changes in retinal thickness, specifically within the inner retinal layers. These changes are indicative of an underlying neurodegenerative process that may precede vascular alterations, potentially offering insights into early disease mechanisms. A significant loss of macular visual function and corresponding thinning of the ganglion cell layer (GCL) has been reported in the pericentral area of the macula in patients with type 1 diabetes mellitus (DM1) and no or minimal DR [5]. Longitudinal studies have also demonstrated that before the onset of DR, DM1 patients experience progressive thinning of the inner retinal layers, including the retinal nerve fibre layer (RNFL), GCL, and inner nuclear layer (INL) [6]. Furthermore, GCL thinning in the pericentral area and RNFL thinning in the peripheral macula have been linked to diabetes duration and minimal DR status, with DR status emerging as the most important explanatory variable [7]. Collectively, these findings reinforce the concept that diabetes exerts an early neurodegenerative effect on the retina, preceding significant vascular involvement.

Studies have shown that some long-term DM patients may never develop DR or other microvascular complications, even after 50 years of disease duration, regardless of glycosylated haemoglobin (HbA1c) levels. Research attributes this to endogenous protective factors. Studies have described that a high proportion of long-term DM patients remain free from proliferative DR (42.6%) and nephropathy (86.9%), with minimal retinopathy progression after decades of disease. Reduced levels of advanced glycation end (AGE) products such as carboxyethyl-lysine and pentosidine are strongly linked to fewer complications, indicating a protective mechanism against vascular damage [8].

Moreover, residual beta-cell function, as indicated by stimulated C-peptide levels, has been associated with reduced risks of complications. In the Diabetes Control and Complications Trial, a 50% increase in C-peptide correlated with a 25% reduction in sustained retinopathy and improved metabolic outcomes, such as lower HbA1c and reduced insulin requirements [9]. These findings suggest that factors like C-peptide preservation and protection against AGE-related damage may help to explain why some individuals resist long-term complications, offering potential targets for therapeutic strategies. It is important to recognize that over an extended period of time, these individuals can show alterations in OCTA and OCT without evident signs of DR [4].

The aim of our study was to evaluate longitudinal microvascular alterations in long-term DM1 patients without signs of DR evaluated by OCTA after a 4-year follow-up period and to correlate these changes with possible modifications to inner retinal thickness. Longitudinal studies on retinal changes in DM patients are limited, with most focusing on type 2 DM (DM2) rather than DM1. To date, very few longitudinal studies have been carried out in DM1 patients without DR. To our knowledge, Vujosevic et al. [10] were the first authors to investigate such changes. These limited findings highlight the lack of data on longitudinal changes in DM1 patients without DR, particularly in older populations with longer disease durations.

## 2. Materials and Methods

A longitudinal study in search of changes in OCTA findings over time in long-term DM1 patients without DR was conducted. The study was performed following the tenets of the Helsinki Declaration and in accordance with Spanish legislation in the field of biomedical research and the protection of personal data (Organic Law 3/2018 and Laws 41/2002 and 14/2007 on biomedical research). The study was approved by the Aragon Clinical Ethics Committee (CEICA PI17/0398 and PI23/063). Patients were evaluated in 2018 and re-evaluated in 2022.

A total of 40 eyes from 40 DM1 patients, diagnosed at least 15 years prior and maintaining stable glycaemic control, were initially included. The study was repeated after 4 years to evaluate any changes. During the follow-up period, 7 were lost (1 due to death, 4 relocations, and 2 missed appointments), 5 developed DR, and 1 was diagnosed with primary open-angle glaucoma. In the follow-up phase, 27 eyes of 27 patients were reassessed.

The inclusion criteria comprised DM1 patients who showed no visible vascular signs of DR, had a disease duration of over 20 years, were under the care of the endocrinology unit, were aged between 18 and 70 years, were of Caucasian ethnicity, and had provided a signed informed consent form for both examinations. Their best-corrected visual acuity (BCVA) was 20/25 or better on the Snellen chart, a spherical equivalent (SE) of less than 6 dioptres (D) with astigmatism less than 3D, and lens opacities graded below 1.0 in the Lens Opacities Classification System III (LOCS III).

The exclusion criteria encompassed the presence of any sign of DR; indications of glaucoma including intraocular pressure (IOP) of 20 mmHg or higher, as measured by Goldmann tonometry; or changes in the optic nerve suggesting glaucoma; and any ophthalmological disease that could impact the study data, including previous ocular surgery or an uncontrolled systemic disease.

Each patient underwent a comprehensive medical history evaluation, including the duration of their DM, glycaemic control measured by their HbA1c levels, DM treatments, arterial blood pressure, and lipid levels.

The ophthalmological examination included BCVA with the Early Treatment Diabetic Retinopathy Study (ETDRS) chart and conversion to the minimal angle of resolution LogMAR units, axial length (AL) with optical biometry using Aladdin KR-1W (Topcon Eye Care Company, Tokyo, Japan), slit lamp biomicroscopy of both anterior and posterior poles, IOP with Goldmann tonometry, ophthalmoscopy examination, wide-field retinography with Clarus 700 (Carl Zeiss Meditec, Dublin, CA, USA), swept-source OCT (SS-OCT), and 3 × 3 mm OCTA image collection with Deep Range Imaging (DRI)-Triton SS-OCT (Topcon Eye Care Company, Tokyo, Japan) with IMAGEnet 6 software version 1.22.1.14101© 2014 (Topcon Corporation, Tokyo, Japan) for both visits. These processes occurred after pupil dilation with Tropicamide^®^ (Alcon Cusi, Barcelona, Spain) studying the retinal superficial capillary plexus (SCP), deep capillary plexus (DCP), and the CC. In the 3 × 3 mm image, the SCP VD was provided by the device in a 3 mm grid with a centre area with a diameter of 1 mm and 4 quadrants in the surrounding ring: superior (S), inferior (I), temporal (T), and nasal (N). The VD represents the area occupied by blood vessels in the box of the SCP (% of positive pixels vs. total pixels in area of interest). For the VD of the other capillary plexuses, the reference lines in the OCT profile were modified in the device to identify the layers occupied by each plexus.

The FAZ metrics (area and diameters) were measured manually using the measuring tool provided by the device. The quality of each image was assessed by two independent observers (MSP, IP). Images with inadequate quality that could interfere with VD evaluation were excluded. The mean of two measured values was considered. We also analysed a 6 × 6 macular cube, checking for correct segmentation and quality (>70/100 without artifacts). Retinal thickness was studied in the different areas provided by the ETDRS grid and expressed in micrometres (µm). The grid was divided into a central area with a 1 mm diameter and 2 surrounding rings, a parafoveal or inner ring (I) with a 3 mm diameter, and a perifoveal or outer ring (O) with a 6 mm diameter. Each of the rings was also divided into 4 quadrants: S, I, T, and N. The central area was named C, and 8 quadrants with an inner or outer (I or O) ring followed (S, I, T, and N).

DRI-Triton OCT was used to examine various retinal protocols: total retinal thickness, spanning from the internal limiting membrane (ILM) to the retinal pigment epithelium (RPE); RNFL; GCL+ protocol, which measures the thickness from the outer limit of the RNFL to the outer limit of the inner plexiform layer (IPL), incorporating the GCL and the IPL; and lastly, the GCL++ protocol, ranging from the ILM to the outer limit of the IPL, which includes the entire ganglion cell complex (RNFL, GCL, and IPL).

### Statistical Analysis

The information for each variable was recorded in a Microsoft Office Excel 2016 database (Microsoft Office Excel 2016, Microsoft Corporation, Redmond, WA, USA). Statistical analyses were performed using the Statistical Package for the Social Sciences software (SPSS version 22.0, SPSS Inc., IBM Corporation, Armonk, NY, USA). A descriptive analysis of the sample with its demographic variables and clinical characteristics was performed. Data normality was analysed using the Kolmogorov-Smirnov test. As the parameters did not adhere to a normal distribution, differences between the two related samples were assessed using the Wilcoxon signed-rank test, and Spearman’s Rho coefficient test was utilized to calculate the bivariate correlations. A value of *p* < 0.05 indicated statistical significance for all analyses.

## 3. Results

In the second phase of the study, 2 out of 27 eyes were excluded after assessing measurement quality, leaving a final sample of 25 eyes from 25 patients. The average age of the participants was 46.84 ± 11.28 years, with an average diabetes duration of 28.88 ± 8.04 years. The mean HbA1c level was 7.60 ± 0.99% at the last examination, which showed a significant increase (*p* = 0.032) from the 2018 level of 7.34 ± 0.94%. Other metabolic values are detailed in Table 1.

In the SCP, VD increased across all areas, reaching statistical significance in all areas except the inferior quadrant. No changes were observed in FAZ metrics. For the DCP, VD significantly increased in the temporal quadrant but decreased in the inferior quadrant, along with a reduction in the FAZ horizontal diameter. The nasal region of the CC showed a significant increase in VD (Table 2).

**Figure 1:**
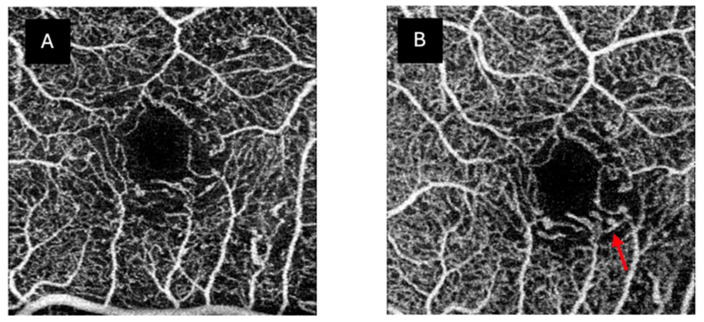
Images from one of the DM1 patients showing changes in the SCP, with an increase in capillary dropout and the emergence of more microaneurysms in the ischaemic area. (**A**) The SCP in 2018; (**B**) the SCP in 2022. The red arrow shows a new microaneurysm.

Morphological findings revealed frequent abnormalities, such as changes in the FAZ morphology (SCP 92%, DCP 88%), microaneurysms in both plexuses (SCP 8%, DCP 20%), and capillary loss (SCP 96%, DCP 96%, CC 76%) (Table 3). Only one patient was considered to have a morphologically normal SCP and DCP. The CC was considered normal in 25% of patients. No morphological differences were noted between time points, except for two patients who showed increased capillary dropout and microaneurysms (Figure 1).

Total retinal thickness and inner retinal layers remained unchanged over the follow-up period. Central retinal thickness values were consistent at both time points. Significant differences were found in the GCL+ protocol in quadrant II (*p* = 0.04), but GCL++ values remained similar (Table 4).

Spearman’s Rho test revealed a negative correlation between disease duration and VD in the temporal quadrant of the SCP at both time points (Table 5 and Table 6). At the initial time point, there was a positive correlation with the central area of the DCP. The total retinal thickness protocol showed a negative correlation with quadrant II at both time points, and with the IN quadrant in 2018. For the GCL+ protocol, there was a negative correlation with all parafoveal quadrants, and for the ganglion cell complex, a negative correlation was noted in the IT and II quadrants in 2018.

In summary, disease duration negatively correlated with VD in the temporal SCP quadrant and was associated with thickness loss primarily in the parafoveal GCL+. HbA1c levels negatively correlated with VD in the S quadrant of both retinal plexuses and the central area of the CC, as well as with all quadrants of the GCL++ protocol, except the OS area.

## 4. Discussion

DR remains the leading cause of preventable blindness in the working-age population in many developed countries. Although traditionally, ocular complications of DM have been considered to be of microvascular origin, there is increasing evidence that a neurodegenerative process takes part prior to the appearance of vascular lesions. This suggests the coexistence of two processes, neuropathic and vascular, as described by Simó and colleagues [11]. DR is a neurovascular disorder [1], and identifying biomarkers present before its development could be beneficial. This neurodegeneration may account for the decrease in VA observed during follow-up.

The aim of our study was to evaluate longitudinal retinal microvascularisation changes in long-term DM1 patients without DR using OCTA. The existence of vascular changes in OCTA in DM patients without visible DR signs has been documented in the literature [4]. However, there are only a few studies where a long-standing DM population without DR lesions has been examined for changes in the VD of the different retinal plexuses over an extended period of time, and the existing studies have mainly been performed in DM2 patients. We aimed to correlate these findings with possible modifications in the inner retina that could imply changes in flow related to the loss of ganglion cells or RNFL thinning.

To our knowledge, Vujosevic et al. [10] were the first authors to examine longitudinal changes in DM1 patients without DR. They followed healthy subjects and DM patients for 3 years. They found changes in the DM2 group, with decreases in the perfusion density and significant thinning in the perifoveal GCL++ ring, decreased perfusion density at the DCP and CC, and increased FAZ in the DCP. They included 20 DM1 patients who were younger than our subjects, with shorter evolution times, but with HbA1c levels similar to our patients; the only finding was an increase in the FAZ perimeter in the DCP. Sacconi et al. also investigated changes in postpaediatric DM1 patients during a 3-year follow-up period [12]. They found impairment of the DCP at the baseline with no changes after follow-up.

Aschauer et al. [13], after a 2-year longitudinal study in DM2 with no DR or mild-moderate DR, found a reduction in SCP perfusion and an increase in FAZ parameters. They did not observe changes in DCP perfusion. In addition, they observed a diminution in GCL and IPL thickness in their patients. Marques et al. [14] also reported a decrease in VD in the SCP and DCP of DM2 patients at all the studied ETDRS levels.

After a 4-year follow-up period, we found an increase in SCP VD in DM1 patients, similar to the description of Vujosevic et al. [10] regarding their DM2 patients. However, we did not identify changes in the inner retinal thickness that could explain a decrease in the blood supply demands at the RNFL or GCL. Retinal vascularization lacks autonomic regulation; therefore, vascularization is primarily controlled by mediators released by retinal and endothelial cells [15]. Blood flow regulation is known to be altered in DM patients, with different studies showing varying results. In individuals within the first 5 years of disease onset, it is common to observe reduced flow with arterial and arteriolar constriction. Feke et al. [16] reported an increase in major artery diameter and decreased blood flow before the onset of DR, likely as a compensatory response to increased resistance in smaller vessels. These findings differ based on the stage of DR [17,18]. Curtis et al. [19] suggested that vascular changes are synergic to the pro-inflammatory retinal status. Tissue damage induces vasodilation and an increase in retinal vascular flow [20], which could be related to different factors affecting vessels [21]. All these studies have used different techniques to evaluate retinal flow, such as fluorescein angiography or Doppler. OCTA provides a comprehensive assessment by evaluating not only VD, but also the FAZ metrics or the adjusted flow index (AFI), allowing us to analyse the impact of blood flow speed on the disease [22]. In general, the AFI tends to decrease as the disease progresses in both the DCP and intermediate capillary plexus. Findings at the SCP level are more controversial. Different studies have reported an increased AFI in DM patients without DR compared to controls that tends to diminish as DR advances [23,24]. Rosen et al. [25] found an increased capillary density in DM patients without DR, demonstrating the potential use of OCTA as a detector of preclinical DR. Zhang et al. also described an increased SCP VD in the DM with no DR group compared to controls. SCP was diminished in in the mild non-proliferative DR group. They demonstrated neurovascular coupling alteration in the retinal plexuses compared to controls [26]. Palochak et al. [27] showed an increase in flow density in DM patients without DR using adaptive optics scanning laser ophthalmoscopy OCT (AO-SLO-OCT). However, this increase diminished after the onset of DR. Onishi et al. [23] proposed that the increase in blood vessels at the SCP might lead to a steal phenomenon in the blood flow of the DCP, contributing to retinal ischaemia, and emphasized the importance of monitoring the changes in the middle capillary plexus, which exhibits alterations similar to those observed in the DCP. The dilation of pre-existing capillary vessels in the SCP could contribute to this increase in VD [22,23]. This hypothesis is sustained by the findings of Vujosevic, who reported an increase in perfusion density [10]. Other factors could also influence these results. Yao and Li described a reduced SCP VD in DM2 patients without clinical DR accompanied by kidney disease. This reduction was in comparison to those without nephropathy, and it was further associated with a decreased peripapillary capillary density [28]. These findings suggest that modifications in SCP flow may serve as biomarkers of microvascular changes preceding the onset of clinical signs of DR. These changes could vary depending on factors such as the type of diabetes, the presence of systemic diseases, patient age, disease duration, and other variables. To comprehensively assess vascular changes in the disease, these variables should be corroborated using other subclinical microvascular markers detectable with OCTA.

We did not evaluate vessel length density; however, Sacconi et al. reported a reduction in vessel length density without changes in the perfusion index. This finding could be a compensatory mechanism involving an increase in the vessel diameter index. Interestingly, these changes were only observed in the DVC, suggesting a weaker self-regulation capacity in this layer [12]. Other authors have also suggested that the DCP has a higher susceptibility to vascular damage [10,29].

Neurovascular coupling has been shown to deteriorate in the initial stages of DM. Changes in retinal vessel dilation induced by flicker stimulation are indicative of flicker-induced retinal vessel alterations and are known to occur in early DM stages, even in the absence of DR [30,31,32]. Our findings support these alterations in retinal vascularization regulation among long-term DM1 patients even without vascular findings, highlighting the capability of OCTA to detect these changes.

Previous research has indicated that longitudinal changes in OCTA values can serve as predictors of DR. Sun et al. [33] demonstrated the ability to predict progression of the FAZ area, VD, and FD of the DCP. However, the SCP metrics were only associated with diabetic macular oedema.

During our 4-year follow-up, we did not observe significant thinning in the retinal layers. The loss of inner retinal neurons in the progression of the disease is a well-established fact and is linked to the neurodegenerative process, as supported by many studies [6,34]. It is likely that a 4-year period might not be enough to detect changes in retinal thickness. The only statistically significant thinning was observed in the II quadrant of the GCL+ protocol. The inferior quadrant was the only region where we did not find differences in VD at the SCP and where VD was reduced in the DCP. This could be attributed to the loss of the GCL layer. Sacconi et al. did not observe any changes in retinal thickness [12]. Other authors have investigated the relationship between macular density and the RNFL, both macular and peripapillary. Hafner et al. found a significant association between the peripapillary RNFL (pRNFL) and parafoveal vascular density. However, they did not find the same correlation with macular RNFL, suggesting that pRNFL could serve as a potential biomarker for the development of DR [35].

We found a negative correlation between disease duration and VD in the T quadrant of the SCP. The temporal area exhibited lower total retinal and GCL++ thicknesses, suggesting it may be the first region where blood flow diminishes after the phase of relative hyperaemia. Disease progression was also associated with a loss of GCL+ thickness in the parafoveal area, where ganglion cell bodies are located. Patients with higher levels of HbA1c showed decreased thickness in the parafoveal area of the GCL+ protocol associated with a decrease in the perifoveal ring of the ganglion cell complex, indicating potential impairment at the RNFL level. These findings underscore the importance of maintaining HbA1c levels below 7% to prevent disease-related deterioration.

A key strength of our study is its focus on long-term DM1 patients, with an average disease duration of 28.88 years. While certain protective factors may mitigate DM complications, their impact varies across renal, ocular, and cardiovascular systems [8,36]. We acknowledge the limitation of our relatively small sample size, primarily due to patient attrition over the follow-up period. This limited sample may reduce the generalizability of our findings and hinder the ability to draw definitive conclusions or perform multiple comparisons. Nevertheless, maintaining long-term follow-up in patients without visible lesions remains challenging. Our evaluation was restricted to 3 × 3 OCTA scans, where the metrics have higher repeatability than larger scan areas [37]. However, this constrained field of view may have caused us to overlook significant vascular changes in the peripheral retina. Peripheral ischemia could play a crucial role in DR development, warranting further investigation. To address this limitation, future studies could consider incorporating wider scan areas, such as 6 × 6 or even 12 × 12 mm fields, to capture a more comprehensive view of the retinal microvascular changes. This broader approach would allow for a better understanding of the peripheral retinal involvement in DR and potentially reveal early signs of ischemia that may not be detected in smaller scans. Moreover, it could help refine diagnostic criteria and provide more precise insights into disease progression, ultimately improving clinical management strategies.

Additionally, the subjective nature of our morphological assessments is a limitation, although we mitigated this by requiring a consensus between two examiners.

In light of our findings, OCTA could serve as a valuable tool for detecting early microvascular changes in diabetic patients, potentially acting as a biomarker for the early stages of DR. By identifying microvascular abnormalities at an earlier stage, OCTA could aid in the timely intervention and monitoring of patients at risk of progression to more severe forms of DR. This could enhance screening strategies, allowing for more personalized care, and enable clinicians to adjust treatment plans proactively. Moreover, early identification of peripheral vascular changes could inform broader management strategies, such as more frequent monitoring or targeted therapies aimed at preventing further microvascular damage. Ultimately, the integration of OCTA into routine clinical practice could lead to more precise and effective management of diabetic eye diseases, improving long-term outcomes for diabetic patients. The combination of multimodal imaging and functional tests may enhance the early detection of DR signs. When integrated with biochemical parameters, these data could provide a valuable approach to predicting the risk of developing DR [38].

## 5. Conclusions

In conclusion, our findings highlight a significant increase in the VD of the SCP in long-term DM1 patients over a 4-year follow-up period, notably without evidence of concurrent thinning of the inner retinal layers. These results suggest that, in the absence of DR, long-term DM1 patients may exhibit distinct microvascular adaptations, potentially indicating protective mechanisms or compensatory responses.

Our study provides valuable insights into the longitudinal retinal changes in DM1 patients, an area of research that remains underexplored compared to studies on DM2. By focusing on a unique patient population with long-standing disease, our findings contribute to a better understanding of early retinal alterations before the onset of DR, which may help to inform future diagnostic and therapeutic strategies. However, further research with larger cohorts and extended follow-up periods is essential to fully elucidate the mechanisms underlying these vascular changes and their potential implications for the progression of DR.

## Figures and Tables

**Table 1:** Mean and standard deviation (SD) of the metabolic characteristics related to the duration and control of the disease and ophthalmic evaluation of the diabetic group at the two studied time points, 2018 and 2022.

Type 1 Diabetes Group	Mean ± SD 2018	Mean ± SD 2022	*p*
Metabolic Values			
Age at diagnosis (years)	17.96 ± 13.43		-
Duration of diabetes (years)	25.68 ± 8.33	28.88 ± 8.04	-
HbA1c (%)	7.34 ± 0.94	7.60 ± 0.99	**0.032**
Glycaemia (mg/dL)	186.60 ± 113.61	160.40 ± 71.21	0.738
Total cholesterol (mg/dL)	194.08 ± 34.95	189.80 ± 35.79	0.204
HDL cholesterol (mg/dL)	57.24 ± 13.78	62.24 ± 12.75	**0.003**
LDL cholesterol (mg/dL)	119.84 ± 27.13	115.00 ± 29.28	0.180
Urea (mg/dL)	35.00 ± 7.67	35.89 ± 11.02	0.831
Creatinine (mg/dL)	0.80 ± 0.12	0.81 ± 0.10	0.474
Albumin/creatinine ratio (mg/g Cr)	7.31 ± 7.83	8.46 ± 12.66	0.475
Ophthalmic Evaluation			
BCVA (LogMAR)	−0.19 ± 0.11	0.04 ± 0.07	**<0.001**
SE (D)	−1.16 ± 1.86	−1.03 ± 1.71	0.302
AL (mm)	23.71 ± 1.12	23.89 ± 1.18	0.954
IOP (mmHg)	16.59 ± 3.00	16.85 ± 2.47	0.635

Statistically significant differences are shown in bold (*p* < 0.05). Abbreviations: HbA1c, glycosylated haemoglobin; HDL, high-density lipoprotein; LDL, low-density lipoprotein; BCVA, best corrected visual acuity; SE, spherical equivalent; D, dioptres; AL, axial length; IOP, intraocular pressure; SD, standard deviation.

**Table 2:** Flow values in the superficial capillary plexus (SCP), deep capillary plexus (DCP), and choriocapillaris (CC) in the diabetic patient group at the two time points of the study (2018, 2022).

		Mean ± SD 2018	Mean ± SD 2022	*p*
SCP	C	19.63 ± 2.66	22.27 ± 3.41	**<0.001**
	S	46.14 ± 3.61	48.14 ± 3.32	**0.005**
	T	44.62 ± 3.38	48.10 ± 2.52	**<0.001**
	N	43.54 ± 2.84	46.68 ± 2.72	**<0.001**
	I	46.81 ± 3.07	47.80 ± 3.08	0.114
	FAZ area	289.00 ± 104.30	288.37 ± 92.84	0.977
	FAZ horizontal Ø	622.16 ± 121.56	609.40 ± 104.03	0.236
	FAZ vertical Ø	576.92 ± 145.57	589.20 ± 136.59	0.206
DCP	C	20.02 ± 4.05	20.30 ± 5.93	0.757
	S	49.29 ± 2.99	48.41 ± 3.18	0.300
	T	45.84 ± 2.75	47.84 ± 3.66	**0.021**
	N	48.06 ± 3.46	47.66 ± 3.68	0.451
	I	51.39 ± 3.19	49.17 ± 3.30	**0.020**
	FAZ area	304.75 ± 86.36	319.36 ± 92.80	0.619
	FAZ horizontal Ø	704.00 ± 135.62	648.32 ± 95.76	**0.007**
	FAZ vertical Ø	572.04 ± 119.38	605.20 ± 116.91	0.146
CC	C	53.85 ± 2.64	54.48 ± 2.23	0.367
	S	51.48 ± 1.96	52.16 ± 2.35	0.313
	T	54.00 ± 1.86	54.28 ± 1.56	0.600
	N	52.74 ± 1.81	53.69 ± 1.50	**0.045**
	I	52.97 ± 2.45	53.17 ± 2.09	0.657

Statistically significant differences are shown in bold (*p* < 0.05). Blood flow was measured as the % of pixels with a positive sign with respect to the total number of pixels in the studied area. Abbreviations: SCP, superficial capillary plexus; DCP, deep capillary plexus; CC, choriocapillaris; C, central; S, superior; N, nasal; T, temporal; I, inferior; FAZ, foveal avascular zone; Ø, diameter.

**Table 3:** Qualitative analysis of the alterations presented in the type 1 diabetes mellitus (DM1) group (%).

	SCP	DCP	CC
FAZ abnormalities	92%	88%	
Marked ischaemia	4%	4%	0%
Capillary dropout	96%	96%	76%
MA	8%	20%	
Normal	4%	4%	24%

Abbreviations: SCP, superficial capillary plexus; DCP, deep capillary plexus; CC, choriocapillaris; FAZ, foveal avascular zone; MA, microaneurysm.

**Table 4:** Mean and standard deviation (SD) of total retina thickness; GCL+ and GCL++ protocol thickness measured in microns (µm) with DRI-Triton SS-OCT in type 1 diabetic patients at the two studied points.

		DM1 2018		DM1 2022		*p*
		Mean	SD	Mean	SD	
Total retina thickness (µm)	C	251.28	23.41	250.16	23.10	0.320
	IS	322.16	15.18	321.60	16.36	0.676
	IT	291.48	62.32	307.12	17.96	0.658
	IN	323.04	14.31	321.92	14.78	0.114
	II	319.72	15.57	318.96	17.01	0.715
	OS	282.08	14.49	282.88	14.92	0.241
	OT	264.44	14.16	264.16	14.01	0.796
	ON	298.84	13.77	297.68	14.65	0.133
	OI	269.28	11.79	271.96	18.81	0.563
GCL+ protocol thickness (µm)	C	47.72	7.57	48.44	6.70	0.234
	IS	95.40	5.18	95.24	8.39	0.078
	IT	90.92	6.51	92.16	9.08	0.311
	IN	94.72	6.98	94.52	10.75	0.218
	II	94.16	7.34	94.04	11.30	**0.042**
	OS	67.56	3.97	67.84	7.80	0.336
	OT	71.80	3.58	72.36	6.01	0.857
	ON	73.56	4.87	74.28	10.29	0.133
	OI	63.76	3.90	64.96	9.22	0.534
GCL++ protocol thickness (µm)	C	52.52	9.95	52.76	9.08	0.678
	IS	123.76	6.29	123.28	10.28	0.777
	IT	111.36	7.93	111.44	9.75	0.415
	IN	120.48	6.95	119.64	9.03	0.977
	II	124.24	7.22	124.04	10.82	0.304
	OS	108.92	7.27	109.32	11.75	0.113
	OT	94.44	4.86	94.36	7.69	0.699
	ON	127.24	6.35	125.24	12.75	0.920
	OI	107.20	5.95	106.56	11.33	0.329

Measurements are divided into the 9 ETDRS areas (abbreviations; C, central; IS, inner superior; IT, inner temporal; II, inner inferior; IN, inner nasal; OS, outer superior; OT, outer temporal; OI, outer inferior; ON, outer nasal). The GCL+ protocol includes the ganglion cell layer and inner plexiform layer, limited from the outer limit of the retinal nerve fibre layer to the inner limit of the inner nuclear layer. The GCL++ protocol includes the retinal nerve fibre layer, ganglion cell layer, and inner plexiform layer, limited from the inner limit of the retinal nerve fibre layer to the inner limit of the inner nuclear layer. Values that reached statistically significant differences are expressed in bold (*p* < 0.05).

**Table 5:** Correlations between the vascular density of both retinal capillary plexuses, choriocapillaris, FAZ area, and diameters with the time of evolution of the disease in both assessed time points and HbA1c at the second assessment time point.

Correlations		Duration of Diabetes (Years)		HbA1c
Spearman’s Rho		1st Assessment	2nd Assessment	2nd Assessment
SCP C	C. coefficient	0.062	−0.114	−0.240
	*p*	0.769	0.586	0.248
SCP S	C. coefficient	−0.294	−0.170	**−0.471**
	*p*	0.153	0.416	**0.017**
SCP T	C. coefficient	**−0.679**	**−0.410**	−0.313
	*p*	**<0.001**	**0.042**	0.128
SCP N	C. coefficient	−0.257	−0.338	−0.174
	*p*	0.215	0.098	0.406
SCP I	C. coefficient	−0.248	−0.352	0.096
	*p*	0.231	0.085	0.650
SCP FAZ AREA	C. coefficient	0.237	0.271	0.272
	*p*	0.254	0.189	0.188
SCP FAZ HOR Ø	C. coefficient	0.234	0.312	0.008
	*p*	0.261	0.129	0.969
SCP FAZ VERT Ø	C. coefficient	0.019	0.155	0.235
	*p*	0.927	0.458	0.258
DCP C	C. coefficient	**0.497**	−0.007	0.087
	*p*	**0.012**	0.976	0.678
DCP S	C. coefficient	−0.180	−0.086	**−0.567**
	*p*	0.389	0.682	**0.003**
DCP T	C. coefficient	−0.167	0.030	−0.077
	*p*	0.426	0.888	0.713
DCP N	C. coefficient	−0.104	−0.043	−0.351
	*p*	0.620	0.840	0.085
DCP I	C. coefficient	0.063	−0.230	0.036
	*p*	0.764	0.269	0.864
DCP FAZ AREA	C. coefficient	−0.078	−0.190	0.031
	*p*	0.710	0.363	0.882
DCP FAZ HOR Ø	C. coefficient	−0.318	−0.042	−0.128
	*p*	0.122	0.843	0.541
DCP FAZ VERT Ø	C. coefficient	−0.065	−0.267	0.183
	*p*	0.757	0.197	0.381
CC C	C. coefficient	0.035	0.071	**−0.517**
	*p*	0.866	0.735	**0.008**
CC S	C. coefficient	−0.070	−0.106	0.055
	*p*	0.739	0.613	0.794
CC T	C. coefficient	−0.127	0.261	−0.123
	*p*	0.547	0.208	0.559
CC N	C. coefficient	−0.205	0.361	−0.313
	*p*	0.325	0.076	0.128
CC I	C. coefficient	−0.077	0.222	0.290
	*p*	0.714	0.286	0.160

Correlations were studied with Spearman’s Rho test. Values that reached statistical significance are expressed in bold (*p* < 0.05). Abbreviations: HbA1c, glycosylated haemoglobin; C. coefficient, correlation coefficient; SCP, superficial capillary plexus; DCP, capillary plexus; CC, choriocapillaris; C, central; S, superior; T, temporal; N, nasal; I, inferior; FAZ, foveal avascular zone; Ø, diameter.

**Table 6:** Correlations between the retinal thickness of total retinal thickness, GCL+ and GCL++ protocols with the time of evolution of the disease in both assessed time points and the HbA1c at the second assessment time point.

Correlations		Duration of Diabetes (Years)		HbA1c
Spearman’s Rho		1st Assessment	2nd Assessment	2nd Assessment
TOTAL RETINAL C	C. coefficient	−0.041	−0.151	0.173
	*p*	0.846	0.471	0.410
TOTAL RETINAL IS	C. coefficient	−0.310	−0.255	−0.029
	*p*	0.131	0.218	0.889
TOTAL RETINAL IT	C. coefficient	−0.114	−0.341	0.123
	*p*	0.588	0.096	0.559
TOTAL RETINAL IN	C. coefficient	−0.425	−0.376	0.091
	*p*	0.034	0.064	0.664
TOTAL RETINAL II	C. coefficient	−0.433	−0.445	0.037
	*p*	0.031	0.026	0.862
TOTAL RETINAL OS	C. coefficient	0.059	−0.041	0.035
	*p*	0.788	0.844	0.869
TOTAL RETINAL OT	C. coefficient	−0.044	−0.208	−0.096
	*p*	0.834	0.319	0.647
TOTAL RETINAL ON	C. coefficient	−0.289	−0.319	−0.069
	*p*	0.161	0.120	0.745
TOTAL RETINAL OI	C. coefficient	−0.205	−0.178	0.037
	*p*	0.325	0.394	0.087
GCL+ C	C. coefficient	−0.051	−0.207	−0.349
	*p*	0.807	0.321	0.087
GCL+ IS	C. coefficient	**−0.433**	**−0.473**	−0.220
	*p*	**0.026**	**0.017**	0.291
GCL+ IT	C. coefficient	**−0.570**	**−0.558**	−0.199
	*p*	**0.003**	**0.004**	0.340
GCL+ IN	C. coefficient	**−0.428**	**−0.444**	−0.263
	*p*	**0.033**	**0.026**	0.203
GCL+ II	C. coefficient	**−0.504**	**−0.439**	−0.131
	*p*	**0.010**	**0.028**	0.532
GCL+ OS	C. coefficient	0.192	−0.052	0.342
	*p*	0.359	0.804	0.095
GCL+ OT	C. coefficient	0.080	−0.341	−0.217
	*p*	0.702	0.095	0.297
GCL+ ON	C. coefficient	−0.058	−0.184	0.219
	*p*	0.784	0.380	0.293
GCL+ OI	C. coefficient	−0.014	−0.075	0.011
	*p*	0.945	0.721	0.960
GCL++ C	C. coefficient	0.001	−0.222	**−0.401**
	*p*	0.996	0.286	**0.047**
GCL++ IS	C. coefficient	−0.374	−0.373	**−0.707**
	*p*	0.065	0.066	**<0.001**
GCL++ IT	C. coefficient	**−0.586**	−0.340	**−0.399**
	*p*	**0.004**	0.096	**0.048**
GCL++ IN	C. coefficient	−0.287	−0.231	**−0.619**
	*p*	0.164	0.266	**0.001**
GCL++ II	C. coefficient	**−0.469**	−0.297	**−0.427**
	*p*	**0.018**	0.149	**0.033**
GCL++ OS	C. coefficient	0.379	0.018	−0.322
	*p*	0.062	0.933	0.117
GCL++ OT	C. coefficient	0.132	−0.309	**−0.493**
	*p*	0.528	0.133	**0.012**
GCL++ ON	C. coefficient	0.022	−0.045	**−0.478**
	*p*	0.917	0.832	**0.016**
GCL++ OI	C. coefficient	0.029	0.058	**−0.435**
	*p*	0.890	0.782	**0.030**

Correlations were studied with Spearman’s Rho test. Values that reached statistical significance are expressed and bold (*p* < 0.05). Abbreviations: HbA1c, glycosylated haemoglobin; C. coefficient, correlation coefficient; C, central; IS, inner superior; IT, inner temporal; II, inner inferior; IN, inner nasal; OS, outer superior; OT, outer temporal; OI, outer inferior; ON, outer nasal; GCL+: ganglion cell layer + inner plexiform layer; GCL++: retinal nerve fibre layer + ganglion cell layer + inner plexiform layer.

## Data Availability

The data presented in this study are available on request from the corresponding author due to patient privacy.

## References

[1] Solomon S.D., Chew E., Duh E.J., Sobrin L., Sun J.K., VanderBeek B.L., Wykoff C.C., Gardner T.W. (2017). Diabetic Retinopathy: A Position Statement by the American Diabetes Association. Diabetes Care.

[2] Williams R., Karuranga S., Malanda B., Saeedi P., Basit A., Besançon S., Bommer C., Esteghamati A., Ogurtsova K., Zhang P. (2020). Global and Regional Estimates and Projections of Diabetes-Related Health Expenditure: Results from the International Diabetes Federation Diabetes Atlas, 9th Edition. Diabetes Res. Clin. Pract..

[3] Williams R., Airey M., Baxter H., Forrester J., Kennedy-Martin T., Girach A. (2004). Epidemiology of Diabetic Retinopathy and Macular Oedema: A Systematic Review. Eye.

[4] Zhang B., Chou Y., Zhao X., Yang J., Chen Y. (2021). Early Detection of Microvascular Impairments with Optical Coherence Tomography Angiography in Diabetic Patients Without Clinical Retinopathy: A Meta-Analysis. Am. J. Ophthalmol..

[5] van Dijk H.W., Verbraak F.D., Stehouwer M., Kok P.H.B., Garvin M.K., Sonka M., DeVries J.H., Schlingemann R.O., Abràmoff M.D. (2011). Association of Visual Function and Ganglion Cell Layer Thickness in Patients with Diabetes Mellitus Type 1 and No or Minimal Diabetic Retinopathy. Vision Res..

[6] Pinilla I., Idoipe M., Perdices L., Sanchez-Cano A., Acha J., Lopez-Galvez M.I., Cuenca N., Abecia E., Orduna-Hospital E. (2020). Changes in total and inner retinal thicknesses in type 1 diabetes with no retinopathy after 8 years of follow-up. Retina.

[7] van Dijk H.W., Verbraak F.D., Kok P.H.B., Garvin M.K., Sonka M., Lee K., Devries J.H., Michels R.P.J., van Velthoven M.E.J., Schlingemann R.O. (2010). Decreased Retinal Ganglion Cell Layer Thickness in Patients with Type 1 Diabetes. Investig. Ophthalmol. Vis. Sci..

[8] Sun J.K., Keenan H.A., Cavallerano J.D., Asztalos B.F., Schaefer E.J., Sell D.R., Strauch C.M., Monnier V.M., Doria A., Aiello L.P. (2011). Protection from Retinopathy and Other Complications in Patients with Type 1 Diabetes of Extreme Duration: The Joslin 50-Year Medalist Study. Diabetes Care.

[9] Lachin J.M., McGee P., Palmer J.P. (2014). Impact of C-Peptide Preservation on Metabolic and Clinical Outcomes in the Diabetes Control and Complications Trial. Diabetes.

[10] Vujosevic S., Muraca A., Alkabes M., Villani E., Cavarzeran F., Rossetti L., De Cilla S. (2019). Early Microvascular and Neural Changes in Patients with Type 1 and Type 2 Diabetes Mellitus without Clinical Signs of Diabetic Retinopathy. Retina.

[11] Simó R., Stitt A.W., Gardner T.W. (2018). Neurodegeneration in Diabetic Retinopathy: Does It Really Matter?. Diabetologia.

[12] Sacconi R., Tombolini B., Cartabellotta A., Zerbini G., Bandello F., Querques G. (2024). Structural and Functional Characterization of Retinal Impairment in T1DM Patients without Diabetic Retinopathy: A 3-Year Longitudinal Study. Acta Diabetol..

[13] Aschauer J., Pollreisz A., Karst S., Hülsmann M., Hajdu D., Datlinger F., Egner B., Kriechbaum K., Pablik E., Schmidt-Erfurth U.M. (2022). Longitudinal Analysis of Microvascular Perfusion and Neurodegenerative Changes in Early Type 2 Diabetic Retinal Disease. Br. J. Ophthalmol..

[14] Marques I.P., Alves D., Santos T., Mendes L., Lobo C., Santos A.R., Durbin M., Cunha-Vaz J. (2020). Characterization of Disease Progression in the Initial Stages of Retinopathy in Type 2 Diabetes: A 2-Year Longitudinal Study. Investig. Opthalmol. Vis. Sci..

[15] Delaey C., van de Voorde J. (2000). Regulatory Mechanisms in the Retinal and Choroidal Circulation. Ophthalmic Res..

[16] Feke G.T., Buzney S.M., Ogasawara H., Fujio N., Goger D.G., Spack N.P., Gabbay K.H. (1994). Retinal Circulatory Abnormalities in Type 1 Diabetes. Investig. Ophthalmol. Vis. Sci..

[17] Clermont A.C., Aiello L.P., Mori F., Aiello L.M., Bursell S.-E. (1997). Vascular Endothelial Growth Factor and Severity of Nonproliferative Diabetic Retinopathy Mediate Retinal Hemodynamics In Vivo: A Potential Role for Vascular Endothelial Growth Factor in the Progression of Nonproliferative Diabetic Retinopathy. Am. J. Ophthalmol..

[18] Grunwald J.E., Riva C.E., Baine J., Brucker A.J. (1992). Total Retinal Volumetric Blood Flow Rate in Diabetic Patients with Poor Glycemic Control. Investig. Ophthalmol. Vis. Sci..

[19] Curtis T.M., Gardiner T.A., Stitt A.W. (2009). Microvascular Lesions of Diabetic Retinopathy: Clues towards Understanding Pathogenesis?. Eye.

[20] Scholfield C.N., McGeown J.G., Curtis T.M. (2007). Cellular Physiology of Retinal and Choroidal Arteriolar Smooth Muscle Cells. Microcirculation.

[21] Delaey C., Boussery K., Van de Voorde J. (2000). A Retinal-Derived Relaxing Factor Mediates the Hypoxic Vasodilation of Retinal Arteries. Investig. Ophthalmol. Vis. Sci..

[22] Nesper P.L., Roberts P.K., Onishi A.C., Chai H., Liu L., Jampol L.M., Fawzi A.A. (2017). Quantifying Microvascular Abnormalities with Increasing Severity of Diabetic Retinopathy Using Optical Coherence Tomography Angiography. Investig. Opthalmol. Vis. Sci..

[23] Onishi A.C., Nesper P.L., Roberts P.K., Moharram G.A., Chai H., Liu L., Jampol L.M., Fawzi A.A. (2018). Importance of Considering the Middle Capillary Plexus on OCT Angiography in Diabetic Retinopathy. Investig. Opthalmol. Vis. Sci..

[24] Ong J.X., Fawzi A.A. (2022). Perspectives on Diabetic Retinopathy from Advanced Retinal Vascular Imaging. Eye.

[25] Rosen R.B., Andrade Romo J.S., Krawitz B.D., Mo S., Fawzi A.A., Linderman R.E., Carroll J., Pinhas A., Chui T.Y.P.P. (2019). Earliest Evidence of Preclinical Diabetic Retinopathy Revealed Using Optical Coherence Tomography Angiography Perfused Capillary Density. Am. J. Ophthalmol..

[26] Zhang Y.S., Mucollari I., Kwan C.C., Dingillo G., Amar J., Schwartz G.W., Fawzi A.A. (2020). Reversed Neurovascular Coupling on Optical Coherence Tomography Angiography Is the Earliest Detectable Abnormality before Clinical Diabetic Retinopathy. J. Clin. Med..

[27] Palochak C.M.A., Lee H.E., Song J., Geng A., Linsenmeier R.A., Burns S.A., Fawzi A.A. (2019). Retinal Blood Velocity and Flow in Early Diabetes and Diabetic Retinopathy Using Adaptive Optics Scanning Laser Ophthalmoscopy. J. Clin. Med..

[28] Yao H., Li Z. (2023). Is Preclinical Diabetic Retinopathy in Diabetic Nephropathy Individuals More Severe?. Front. Endocrinol..

[29] Nakahara T., Hoshino M., Hoshino S., Mori A., Sakamoto K., Ishii K. (2015). Structural and Functional Changes in Retinal Vasculature Induced by Retinal Ischemia-Reperfusion in Rats. Exp. Eye Res..

[30] Nippert A.R., Newman E.A. (2020). Regulation of Blood Flow in Diabetic Retinopathy. Vis. Neurosci..

[31] Mozolewska-Piotrowska K., Nowacka M., Masiuk M., Świder M., Babiak K., Safranow K., Machalińska A. (2019). Flicker-Induced Retinal Vessels Dilatation in Diabetic Patients without Clinically Detectable Diabetic Retinopathy. Klin. Oczna.

[32] Lim L.S., Ling L.H., Ong P.G., Foulds W., Tai E.S., Wong T.Y. (2017). Dynamic Responses in Retinal Vessel Caliber with Flicker Light Stimulation and Risk of Diabetic Retinopathy and Its Progression. Investig. Opthalmol. Vis. Sci..

[33] Sun Z., Tang F., Wong R., Lok J., Szeto S.K.H., Chan J.C.K., Chan C.K.M., Tham C.C., Ng D.S., Cheung C.Y. (2019). OCT Angiography Metrics Predict Progression of Diabetic Retinopathy and Development of Diabetic Macular Edema. Ophthalmology.

[34] Nathan D.M. (2014). The Diabetes Control and Complications Trial/Epidemiology of Diabetes Interventions and Complications Study at 30 Years: Overview. Diabetes Care.

[35] Hafner J., Karst S., Sacu S., Scholda C., Pablik E., Schmidt-Erfurth U. (2019). Correlation between Corneal and Retinal Neurodegenerative Changes and Their Association with Microvascular Perfusion in Type II Diabetes. Acta Ophthalmol..

[36] Oram R.A., Jones A.G., Besser R.E.J., Knight B.A., Shields B.M., Brown R.J., Hattersley A.T., McDonald T.J. (2014). The Majority of Patients with Long-Duration Type 1 Diabetes Are Insulin Microsecretors and Have Functioning Beta Cells. Diabetologia.

[37] Chen F.K., Menghini M., Hansen A., Mackey D.A., Constable I.J., Sampson D.M. (2018). Intrasession Repeatability and Interocular Symmetry of Foveal Avascular Zone and Retinal Vessel Density in OCT Angiography. Transl. Vis. Sci. Technol..

[38] Zhang Z., Deng C., Paulus Y.M. (2024). Advances in Structural and Functional Retinal Imaging and Biomarkers for Early Detection of Diabetic Retinopathy. Biomedicines.

